# Neuroprotective Effect of Ginseng against Alteration of Calcium Binding Proteins Immunoreactivity in the Mice Hippocampus after Radiofrequency Exposure

**DOI:** 10.1155/2013/812641

**Published:** 2013-08-29

**Authors:** Dhiraj Maskey, Jin-Koo Lee, Hak Rim Kim, Hyung-Gun Kim

**Affiliations:** Department of Pharmacology, College of Medicine, Dankook Translational Research Center, Dankook University, 119 Dandaero, Anseo-dong, Dongnam-gu, Cheonan, Chungnam 330-714, Republic of Korea

## Abstract

Calcium binding proteins (CaBPs) such as calbindin D28-k, parvalbumin, and calretinin are able to bind Ca^2+^ with high affinity. Changes in Ca^2+^ concentrations via CaBPs can disturb Ca^2+^ homeostasis. Brain damage can be induced by the prolonged electromagnetic field (EMF) exposure with loss of interacellular Ca^2+^ balance. The present study investigated the radioprotective effect of ginseng in regard to CaBPs immunoreactivity (IR) in the hippocampus through immunohistochemistry after one-month exposure at 1.6 SAR value by comparing sham control with exposed and ginseng-treated exposed groups separately. Loss of dendritic arborization was noted with the CaBPs in the Cornu Ammonis areas as well as a decrease of staining intensity of the granule cells in the dentate gyrus after exposure while no loss was observed in the ginseng-treated group. A significant difference in the relative mean density was noted between control and exposed groups but was nonsignificant in the ginseng-treated group. Decrease in CaBP IR with changes in the neuronal staining as observed in the exposed group would affect the hippocampal trisynaptic circuit by alteration of the Ca^2+^ concentration which could be prevented by ginseng. Hence, ginseng could contribute as a radioprotective agent against EMF exposure, contributing to the maintenance of Ca^2+^ homeostasis by preventing impairment of intracellular Ca^2+^ levels in the hippocampus.

## 1. Introduction

Ginseng, the root of Panax ginseng C.A. Meyer, is a widely used herbal medicine with numerous efficacious effects but possessing very low rate of side effects, thereby becoming one of the top-selling natural remedies plus the most popular dietary supplement [1]. Ginseng plays an important role in the central nervous system (CNS) [2, 3] proving to be effective in the attenuation of learning deficits due to brain damage and aging in humans and animals [4–7]. The beneficial effects of the ginseng root on learning and memory are often attributed to ginsenoside Rb1 (Rb1), which enhances the stimulatory effect of neurite outgrowth [8]. Studies suggest that Rb1 protects hippocampal neurons against either ischemia [9] or glutamate-induced neurodegeneration [10], establishing ginseng as a neuroprotective agent against various experimental traumatic brain injuries leading to the possibility of its effectiveness against electromagnetic field (EMF) radiofrequency (RF) injury in the hippocampal subfields.

Rapid advancement in the field of telecommunications has led to dramatic increase in the use of mobile phone technology, generating interest in the biological effects and possible health outcomes of electric and magnetic fields. The extensive use of mobile phones raises questions of their possible biological effects [11] due to EMF exposure, particularly on the CNS. Frequent mobile users have constantly complained of headaches, heat sensation during extended periods of communication [12], and sleep disturbances [13]. Given the close proximity of the mobile device to the brain, higher specific absorption rates (SAR) occur in this part of the body as compared with other parts of the body [14]. The influence of RF in neuronal functions, including regulation of synaptic plasticity, neurotransmitter release, neuronal survival, and learning and memory, has also been reported [15]. Recently, loss of pyramidal cells in the CA areas as well as the dentate gyrus has also been reported [16–18], mainly instigated by studies of possible RF exposure effects on animal models. Despite several biological, epidemiological, and toxicological studies, the potential adverse effects of RF exposure on the CNS are still controversial [19].

Alteration of intracellular signaling pathway via changes in ionic distribution or membrane fluidity could be influenced by EMF [14]. Physiological calcium (Ca^2+^) entry into neurons regulates normal neuronal development, metabolism, and ageing and is involved in the control of synaptic transmission and its long-term modulation [20] with elevated Ca^2+^ levels leading to neuronal degeneration [21]. Radiofrequency (RF) exposure can induce Ca^2+^ efflux from the brain and isolated neurons [22–24]. Ca^2+^ homeostasis in the brain is regulated by influx and efflux systems but is also affected by calcium binding proteins (CaBPs). 

Ca^2+^ plays a complex role as an important moderator of a number of vital physiological processes like neuronal excitability, axonal transport, synthesis and release of neurotransmitters, membrane permeability, and enzyme activities [25]. Neuronal Ca^2+^ might play a role in neuron survival as well as programmed cell death and pathological neuronal degeneration with disturbance in Ca^2+^ regulation leading to lethal effects [26]. Therefore, efficient regulation of intracellular free Ca^2+^concentration is crucial for neuronal function and survival, which can be achieved by an active uptake mechanism of the cell's internal membrane structures such as mitochondria and endoplasmic reticulum as well as CaBPs. Considering the importance of Ca^2+^ homeostasis in numerous processes for cell viability, including neurons, and modulation of intracellular Ca^2+^ concentration, CaBPs such as calbindin D28-k (CB), parvalbumin (PV), and calretinin (CR), belonging to the EF-hand type, could play a crucial role in the regulation of Ca^2+^ homeostasis by buffering the intracellular Ca^2+^concentration and transporting Ca^2+^ [27]. A neuroprotective role with a sole function as a cytoplasmic Ca^2+^ buffer has been postulated for CaBPs, the impairment of which leads to the failure of buffering of the intracellular Ca^2+^ and neuronal death [28]. CaBP characterizes specific neuronal types in the CNS [29] among which PV, CB [30], and CR [30] are considered to be excellent neuronal markers for a subpopulation of the hippocampal neurons. Loss of CaBPs has been linked with impaired Ca^2+^ homeostasis and is related with both neuronal and behavioral deficits [31]. Hence, dysregulation of Ca^2+^ homeostasis could contribute to impaired memory processes of the hippocampus. Characterization of CaBPs is important as each neuron may be associated with particular functional properties, loss of which could lead to different manifestations.

Considering the reported effect of purified ginseng components as radioprotective agents in irradiated rodents [10, 32] and CaBP implicated as an important regulator of pathological neuronal degeneration, these two factors could be used to measure the damage due to RF exposure and to observe the beneficial effect ginseng might have as a radioprotective agent in the hippocampal subfields against RF injury. Hence the aim of the present study was to investigate the effect of ginseng in the CaBP IR in the different hippocampal subfields following 835 MHz RF exposure at SAR 1.6 W/kg for 1 month.

## 2. Materials and Methods

### 2.1. Red Ginseng Extracts

 Red Ginseng extracts (RG) manufactured by Korea Ginseng Corporation (Seoul, Republic of Korea) were used in all experiments. RG was made by steaming fresh roots of 6-year-old *P. ginseng* plants at 90°C to 100°C for 3 hr and subsequent drying at 50°C to 80°C. RG was extracted seven times with distilled water at 85°C for 8 hr followed by cooling. RG contained 0.52 mg/g of major ginsenoside-Rg1, 4.03 mg/g of -Rb1, 2.89 mg/g of -Rg3(s), 1.18 mg/g of -Re, 1.98 mg/g of -Rc, 1.97 mg/g of -Rb2, 1.51 mg/g of -Rd, and other minor ginsenosides.

### 2.2. Animals

Six-week-old 20–30 g ICR (Orient Bio Inc.) male mice (*n* = 30) were used for the experiment. Upon arrival, animals were randomized and housed six per cage under the condition of 20 to 25°C. Food and water were supplied ad libitum. Mice were acclimated for one week. All animal procedures were performed according to the National Institutes of Health guidelines of animal research and were approved by Dankook University Institutional Animal Care and Use Committee (DUIACUC), which adheres to the guidelines issued by the Institution of Laboratory of Animal Resources (ILAR). Before conducting the study, mice were categorized into three groups (*n* = 10): (A) sham control (SC), (B) exposed (E1.6), and (C) exposed treated with 30 mg/kg of RG (G1.6).

### 2.3. Radiofrequency Exposure System

The exposure system (Wave Exposer V20) has been described in detail [18]. Briefly, a Wave Exposer V20 emitting 835 MHz equivalent to the Korean Code Division Multiple Access (CDMA) mobile phone frequency was designed by the Division of Information Technology Engineering, Soonchunhyang University. SAR (specific absorption rate) was controlled from 1.6 to 4.0 W/kg, which is the same value as electric field intensity between 59.56 and 94.18 V/m for muscle (=0.92, =57, and =1020 Kg/m^3^) on 835 MHz CDMA frequency. Waves were generated and amplified in an electronic unit and eventually were radiated by a pyramidal rectangular horn antenna connected by a waveguide to coaxial transition. A standard mouse cage of 22 inches was used for the apparatus. Output powers of horn antenna of the exposure apparatus are 2.5 W for SAR 1.6 W/kg and 6.3 W for SAR 4.0 W/kg. Electric field intensities due to SAR values can be calculated, and power value was obtained by a computer simulation with HFSS (High Frequency Structure Simulator) manufactured by Ansoft Co. (Pittsburgh, PA, USA). 5 cylinder-shaped models of mice were used for simulation. The simulation variable was both the mice location and the distance form horn aperture for freely moving mice. Power was obtained by averaging the simulated peak electric field intensities on each mouse body. The wave exposure from horn antenna to the mouse cage was provided by wave absorption material (TDK ceramic absorber) mimicking the radiation exposure in the open environment, which limits the influence the number of mice might have on exposure. The exposure apparatus provides an automatic light system and air conditioning system with a water feeder, with no restriction in movement during exposure eliminating stress during exposure.

### 2.4. Experimental Design and Exposure Condition

For the RF exposure experiment, the entire body of mice was exposed to 835 MHz radiation for one month with average SAR of 1.6 W/kg by using Wave Exposer V20 instrument. The RG extract solution was prepared in a solvent consisting of 0.9% NaCl and 4% Tween 80 and was given orally before exposure according to individual body weight. The exposure was conducted for five hours per day for 30 consecutive days for each group. Three hours after the final exposure in the 30th day, animals were anesthetized with diethyl ether, and their brains were collected using perfusion and fixation procedure with phosphate buffer saline and 4% paraformaldehyde solution.

### 2.5. Immunohistochemical Analysis

Brains were removed from the skull and were postfixed in paraformaldehyde for 24 hours. Then brain tissues were cryoprotected after soaking in a series of sucrose solution (10%, 20%, and 30%) at 4°C until they sank. Serial coronal section, 40 *μ*m, was cut with freezing, sliding microtome and collected in wells. Three separate experiments with all three groups simultaneously of immunohistochemistry were performed with the free floating method. Polyclonal anti-rabbit CB (AB1778; Millipore, CA, USA), polyclonal anti-rabbit PV (AB15736; Millipore, CA, USA), and polyclonal anti-goat CR (AB1550; Millipore, CA, USA) were applied to brain sections at dilution ratios of 1 : 5,000, 1 : 10,000, and 1 : 15,000, respectively, in phosphate buffer saline based blocking buffer containing 1% bovine serum albumin, 0.3% Triton X-100, and 1% normal horse serum. Sections were incubated for 48 hours at 4°C. After three washes with phosphate buffer saline, sections were incubated with the biotinylated secondary antibodies at a dilution ratio of 1 : 250 for 1.5 hours at room temperature. Following additional washes, sections were mounted on gelatin coated slides, dehydrated in ethanol, cleared in xylene, and cover-slipped with DPX. 

### 2.6. Image and Statistical Analysis

Analysis was performed under Olympus BX 51 microscope, and pictures of the sections were taken by a microscope digital camera system (DP50, Olympus, Japan). The NIH image program (Scion Image) was used to determine staining densities. The sum of the gray values of all pixels in a selected region was divided by the total number of pixels in the selected region to determine the mean density of immunoreactivity per unit area (mm^−2^). Mathematically, Mean Density = Immunoreactivity/Area (mm^−2^). Data are expressed as mean ± SD. Comparison of the mean density of the different subfields of the hippocampus (CA1, CA3, and dentate gyrus) between the sham group and exposed groups was done individually by unpaired Student's *t*-test. Differences were considered significant at *P* < 0.05. 

## 3. Results

### 3.1. Histomorphometric Observations

#### 3.1.1. Calbindin D28-k Immunoreactivity

**Figure 1:**
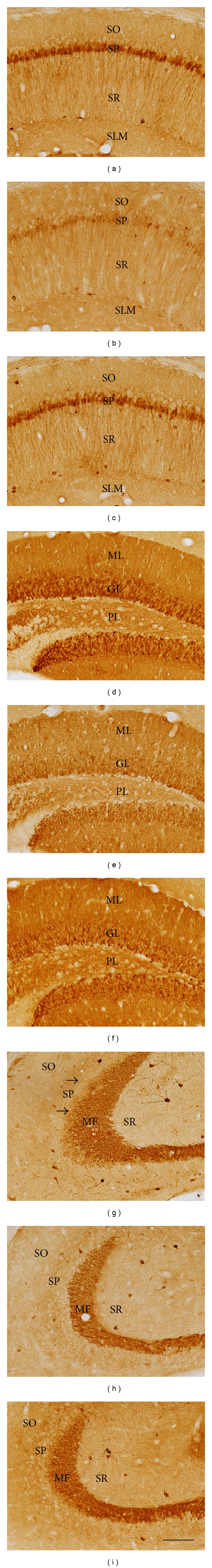
Photomicrograph of calbindin D28-k (CB) immunoreactivity (IR) in coronal sections through the hippocampal subfields of sham control (SC) (a, d, g), radiofrequency-exposed (E1.6) (b, e, h), and ginseng-treated (G1.6) (c, f, i) groups. CA1 (a–c) and CA3 areas (g–i) show various CB-positive neurons. In the various layers of CA1, CB IR pyramidal (thin arrows) and other interneuron (thick arrows) cells were noted areas in all three types (a–c). Strong labeling of the granule cells and mossy fibers (MF) was detected in the dentate gyrus which was highly decreased in E1.6 (f). CA3 area of SC revealed few CB immunopositive neurons in the SR of all three types (g–i). SO: stratum oriens; SP: stratum pyramidale; SR: stratum radiatum; SLM: stratum lacunosum moleculare; SL: stratum lucidum; ML: molecular layer; GL: granular layer; PL: polymorphic layer. Scale bar = 100 *μ*m.

**Figure 2:**
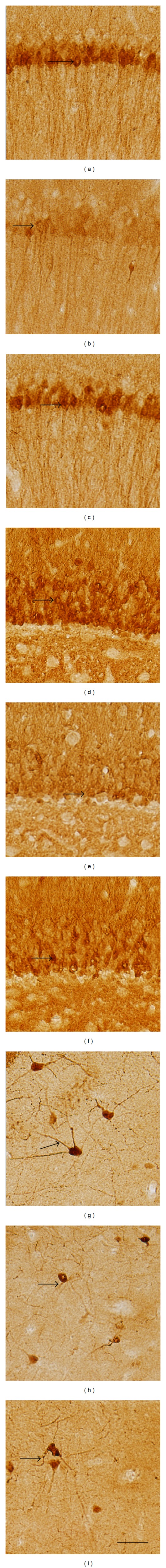
Magnified image of calbindin D28-k (CB) immunoreactivity (IR) in the subfields of sham control (SC) (a, d, g), radiofrequency-exposed (E1.6) (b, e, h), and ginseng-treated (G1.6) (c, f, i) groups. Loss of staining in the soma (arrows) as well as the dendrites of the neurons in the CA1 area of E1.6 was observed (c). GL of E1.6 (e) displayed weakly labeled granule cells (arrows) as compared with SC (d). Granule cells were highly stained in G1.6 (f). The CA3 area revealed darkly stained neurons with dendrites (arrow) arborizing out, which were noted in SC (g) and G1.6 (i). However, E1.6 (h) displayed very faintly labeled neurons lacking dendritic (arrow) arborizations (i). Scale bar = 50 *μ*m.

CA1 and CA3 subfields and the dentate gyrus of all three groups showed presence of CB-positive neurons (Figures 1(a)–1(i)). Various layers of CA1 area like stratum lacunosum (SL), stratum radiatum (SR), and stratum oriens (SO) of all the three groups displayed CB immunostained pyramidal cells bodies and scattered multipolar neurons (Figures 1(a)–1(c)). Few faintly CB-positive cells were present in the stratum pyramidale (SP) of the CA1 area. Intensely labeled single neurons were noted in the SR/SL region of the CA1 area of the SC and G1.6 groups, which were characterized by arborized slender processes, most likely corresponding to the interneurons (Figure 2(a)). However, the neurons in the same region of the E1.6 displayed faint staining along with a lack of dendrites (Figure 2(b)). Highly stained cells with fine dendritic arborization were observed in the SR of CA3 area of both SC and G1.6 groups (Figures 2(g) and 2(i)), while the same region of E1.6 consisted of very faintly labeled cells which lacked dendritic arborization (Figure 2(h)). 

Dentate gyrus (DG) of all three groups displayed the presence of CB IR very distinctly. Granular layer (GL) was highly stained as compared with molecular layer (ML) and polymorphic layer (PL) in all the three groups (Figures 1(d)–1(f)). Perceivable decrement of CB IR was observed in the DG of E1.6 (Figure 1(e)) while such decrement was not present in the G1.6 as compared with SC (Figure 1(f)). Cell bodies and dendrites of granule cells were the main center for localization of CB IR in the DG along with the mossy fibers of PL, which could be followed projecting towards the stratum lucidum (SL) of the CA3 areas (Figures 1(d)–1(f)). Very weak CB IR was noted in the granule cells of E1.6 as compared with SC while such a difference was not observed between SC and G1.6 (Figures 2(d)–2(f)).

#### 3.1.2. Parvalbumin Immunoreactivity

**Figure 3:**
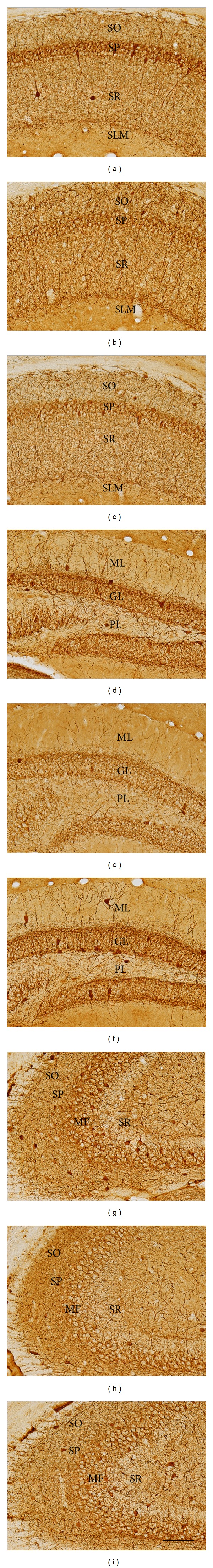
Photomicrograph of parvalbumin (PV) immunoreactivity (IR) in coronal sections through the hippocampal subfields of sham control (SC) (a, d, g), radiofrequency-exposed (E1.6) (b, e, h), and ginseng-treated (G1.6) (c, f, i) groups. Significant decrement of PV IR was observed in the E1.6 when compared with SC and G1.6 (a–i). PV immunopositive neurons were observed in SO and SP layers (a–c) which were very faintly labeled in E1.6 (c). PV immunoreactive fibers were noted to be running perpendicularly in SR. Various highly PV positive neurons along with dendrites running through the ML were observed in the GL of DG. A marked loss of IR was noted in the granular layer of E1.6 (e) which was not noted in G1.6 (f). CA3 area revealed various PV immunoreactive fibers along with few neurons distributed throughout the subfields. Decrease in IR was noted in E1.6 (h) as compared with SC (g) and G1.6 (i). SO: stratum oriens; SP: stratum pyramidale; SR: stratum radiatum; SLM: stratum lacunosum moleculare; SL: stratum lucidum; ML: molecular layer; GL: granular layer; PL: polymorphic layer. Scale bar = 100 *μ*m.

**Figure 4:**
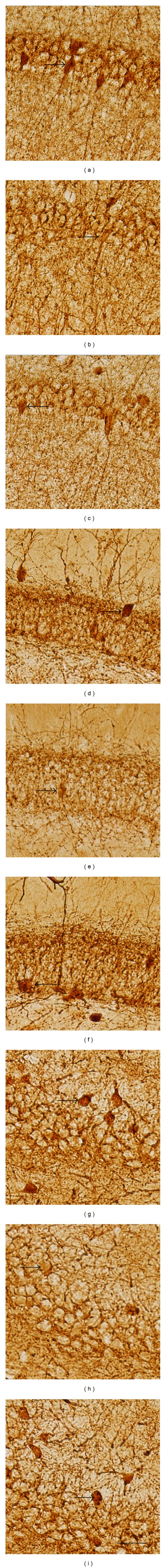
Magnified image of parvalbumin (PV) immunoreactivity (IR) in the subfields of sham control (SC) (a, d, g), radiofrequency-exposed (E1.6) (b, e, h), and ginseng-treated (G1.6) (c, f, i) groups. Neurons (arrows) present in the CA1 area of E1.6 (b) showed decrease in PV IR, which appeared to be very highly labeled in SC (a). Similarly, the staining intensity of the neurons in the granular layer was highly decreased in E1.6 (e) along with its axons (arrows) as compared with SC (d) and G1.6 (f). CA3 area also revealed loss of IR in the neurons (arrows) as well as the dendrites of E1.6 (h). Scale bar = 50 *μ*m.

Wide distribution of PV IR was noted in the hippocampal region throughout the CA1 and CA3 areas as well as in the dentate gyrus. SO of CA1 area displayed consisted of various multipolar and bipolar cells, which were also seen in the SP (Figures 3(a)–3(c)). The SP of the CA1 area displayed PV immunopositive neurons, consisting of long thin axons running through the SR (Figures 3(a) and 4(a)). Severe decrement of PV IR in the neurons of E1.6 was noted (Figures 3(b) and 4(b)) but was not so severe in G1.6 as compared with SC (Figures 3(c) and 4(c)). Perpendicularly running PV immunoreactive fibers were noted in the SC and G1.6 (Figure 3(c)) but were noted to be decreased in E1.6 (Figure 3(b)). Highly PV immunoreactive neurons and processes were noted to be distributed in all the subfields of the CA3 area of SC and G1.6 (Figures 4(g) and 4(i)) but were significantly decreased in E1.6 (Figure 4(h)).

PV IR was detected in all three layers of the dentate gyrus as well as in the mossy fibers of all the three groups to a varying degree (Figures 3(d)–3(f)). The granular layer showed highly immunapositive neurons while the PL consisted of lightly stained neurons. The neurons in the GL were of fusiform, triangular, or stellate shape and gave out dendrites that appeared to be running into the ML as well as the PL (Figures 4(d)–4(f)). Compared with SC, no such loss of PV IR was noted in the neurons of GL of G1.6 (Figure 4(f)) but appeared to highly decrease in the E1.6. Very faintly stained axons were noted to be running through the ML (Figure 4(e)).

#### 3.1.3. Calretinin Immunoreactivity

**Figure 5:**
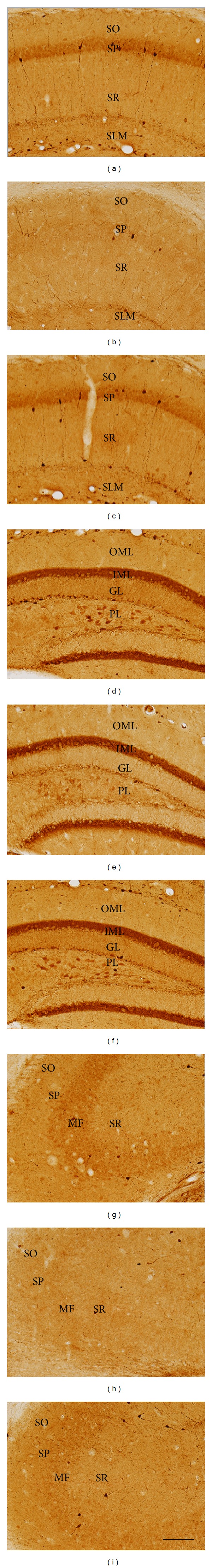
Photomicrograph of calretinin (CR) immunoreactivity (IR) in coronal sections through the hippocampal subfields of sham control (SC) (a, d, g), radiofrequency-exposed (E1.6) (b, e, h), and ginseng-treated (G1.6) (c, f, i) groups. CA1 area consisted of strong CR immunoreactive neurons in SO and SLM along with thin immunoreactive fibers. Severe loss of neuronal staining as well as CR IR was noted in the CA1 of E1.6 (b). Intense CR IR was detected in the IML with highly stained infragranular neurons (d–f). Intensely labeled immunoreactive fibers with few neurons appeared to be present in the CA3 area. Severe decrease of IR was noted in the E1.6 (h) as compared with SC (g) and G1.6 (i). SO: stratum oriens; SP: stratum pyramidale; SR: stratum radiatum; SLM: stratum lacunosum moleculare; SL: stratum lucidum; OML: outer molecular layer; IML: inner molecular layer; GL: granular layer; PL: polymorphic layer. Scale bar = 100 *μ*m.

**Figure 6:**
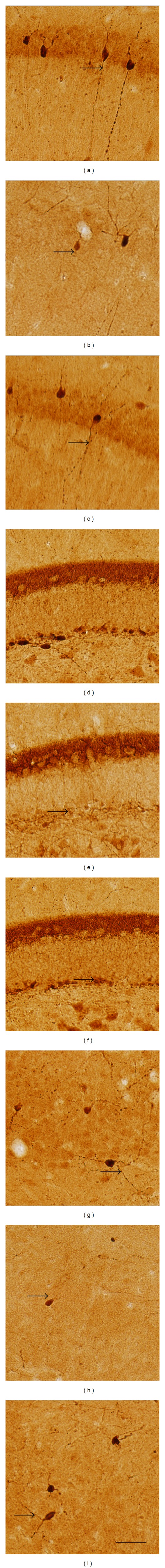
Magnified image of calretinin (CR) immunoreactivity (IR) in the subfields of sham control (SC) (a, d, g), radiofrequency-exposed (E1.6) (b, e, h), and ginseng-treated (G1.6) (c, f, i) groups. Note the loss of staining in axons (arrows) in the CA1 area of the E1.6 (b) which appear to be intact in SC (a) and G1.6 (c). Decrease in the staining intensity as well as severe loss of infragranular neurons (arrows) was noted in the dentate gyrus of E1.6 (e) as compared with SC (d) and G1.6 (f). CR immunopositive neurons displayed intense dendritic arborization (arrows) in the CA3 area of SC (g) and G1.6 (i), but this was absent in E1.6 (h). Scale bar = 50 *μ*m.

Layer specific alterations of CR IR were observed in the CA areas as well as the DG. CA1 and CA3 areas showed CR IR in the subgranular layer along with scattered interneurons. Strongly labeled scattered bipolar, multipolar, and pyramidal neurons were noted in the SL, SR, and SO of the CA1 areas (Figures 5(a)–5(c)). Numerous highly stained cells with axonal arborization were observed in the SO, SP, and SLM while a dense plexus of thin IR fibers was also prominent in the SR of CA1 in all three groups (Figures 5(a)–5(c)). Loss of axonal and dendritic arborization of the neurons in SP of E1.6 showed loss of staining in the axons and dendritic arborization of E1.6 as compared with SC and G1.6 (Figure 6(c)). A similar pattern of CR IR was noted in the CA3 area of all the three groups with varying intensity (Figures 6(g)–6(i)). CA3 of SC and G1.6 showed scattered single neurons with numerous dendritic arborization along with highly stained immunoreactive fibers (Figure 6(g)) while E1.6 displayed loss of dendritic arborization and a decrease in the staining intensity of the immunoreactive fibers with severe decrement of stained neurons (Figures 6(g)–6(i)).

Intense CR IR was noted in the neuropil of inner molecular layer (IML) with moderate staining in the other layers. Strongly immunoreactive infragranular neurons were observed in the SC (Figure 5(d)) and G1.6 (Figure 5(e)) illustrating intense dendritic arborization presumably passing to the outer molecular layer (OML) through the CR immunopositive IML of DG. Infragranular neurons of E1.6 displayed loss of staining intensity (Figure 6(e)). The hilus of the DG of the SC and G1.6 showed intense CR IR dendritic plexuses, along with some stained neurons, but these were prominently decreased in E1.6 (Figures 5(d)–5(f)).

### 3.2. Immunoreactivity Analysis

#### 3.2.1. CB Immunoreactivity

**Figure 7:**
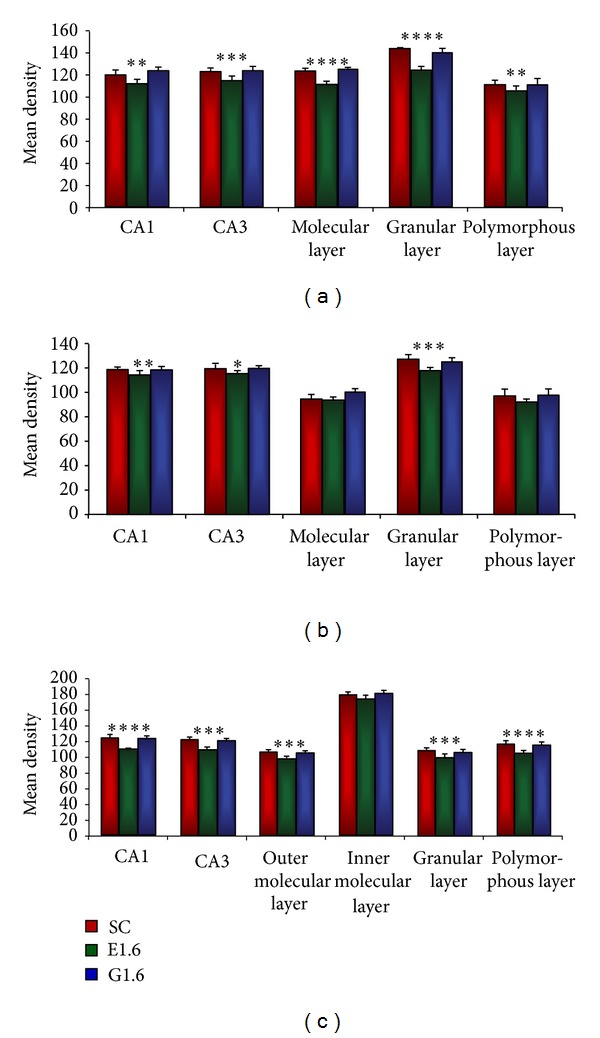
Image analysis of relative density of calbindin D28-k (a), parvalbumin (b), and calretinin (c) immunoreactivity (IR) changes in the hippocampal region (CA1, CA3, and dentate gyrus) of sham control (SC) (a, d, g), radiofrequency-exposed (E1.6) (b, e, h), and ginseng-treated (G1.6) (c, f, i) groups. Significant decrease in the IR was noted in E1.6 in the various different subfields of the hippocampus as compared with SC. The data shown are the mean ± SD values obtained from three different experiments. *P < 0.05, **P < 0.01, ***P < 0.001, and ****P < 0.0001, compared with SC.

Image analysis assessment of the mean density distribution of CB IR in all subfields of the hippocampus (Figure 7(a)) displayed the highest levels in the GL while the lowest was noted in the PL area in all the three groups. CB IR in all the hippocampal subfields was noted to be greatly reduced in the E1.6 as compared with other groups.

E1.6 showed significant difference of IR in CA1 (*P* < 0.01), CA3 (*P* < 0.001), ML (*P* < 0.0001), GL (*P* < 0.0001), and PL (*P* < 0.01) as compared with SC (Figure 7(a)). Comparison of G1.6 with SC showed significant difference only in the GL (*P* < 0.01). No significant difference was noted in the CA1, CA3, ML, and PL areas of G1.6 when compared with SC (Figure 7(a)).

#### 3.2.2. PV Immunoreactivity

In the assessment of image analysis, the relative mean density of different subfields of the hippocampal regions was calculated in order to compare the PV IR distribution between the SC, E1.6, and G1.6 groups. PV IR was observed to be significantly lower in all the areas of the hippocampal formation of the E1.6 than those of SC (Figure 7(b)). All the three groups displayed the highest PV IR in the GL of the DG, and the lowest was observed in the ML and PL areas. 

Significant difference was observed in the CA1 area (*P* < 0.01), CA3 area (*P* < 0.05), and GL (*P* < 0.001) of the E1.6 when compared with SC (Figure 7(b)). Similarly, comparison between SC and G1.6 revealed significant difference in the ML area (*P* < 0.01) while the CA1, CA3, GL, and PL areas did not show any significant difference between the SC and G1.6 groups (Figure 7(b)).

#### 3.2.3. CR Immunoreactivity

Relative mean density analysis was performed to measure the CR IR in the hippocampal subfields of all the groups (Figure 7(c)). The highest CR IR was observed in the IML, whereas the lowest was noted in the OUL in all the three groups. The CR IR was less in all the subfields of the hippocampus of the E1.6 as compared with SC (Figure 7(c)). When compared with SC, E1.6 showed significant changes in the CA1 (*P* < 0.0001), CA3 (*P* < 0.0001), OML (*P* < 0.001), GL (*P* < 0.001), and PL (*P* < 0.0001) (Figure 7(c)). No significant difference was noted between the SC and G1.6 groups in all the subfields of the hippocampus (Figure 7(c)). 

## 4. Discussion

In the present study, we evaluated the CaBP IR in the hippocampal subfields to examine the radioprotective effects of the RG extract against RF exposure at 835 MHz at SAR 1.6 W/kg for one month at 5 hr/day. The study provided results showing the protective effects of RG against EMF exposure, demonstrating that RG could be useful as a radioprotective agent in the CNS.

CaBPs like CB, PV, and CR have an important role in maintaining intracellular Ca^2+^ homeostasis, and its specific distribution pattern in the CNS suggests its involvement in important neuronal activities. CB is associated with regulation of intracellular Ca^2+^and is implicated as a neuronal population marker [33]. PV is known to buffer intracellular Ca^2^, contributing to the maintenance of synaptic properties [34], while CR plays a passive buffering role limiting the rise of intracellular free Ca^2+^ [35]. Alteration in the expression of CaBP may lead to pathological conditions and neurogenerative conditions due to its Ca^2+^ buffering capacity which could influence diverse functions, such as segregating signaling pathways by limiting calcium diffusions [36]. Ca^2+^ is an important component of normal cellular functions and mediates most of the physiological effects triggered by EMF and the ions that are liberated from their intracellular stores. Interactions of Ca^2+^ at the cell membrane have been identified as the first link in the bioeffects from RFR. Release of the neurotransmitter that transfers signals between neurons requires a prerequisite of programmed flow of Ca^2+^ ions through the membranes. Disruption leading to Ca^2+^ leakage would increase the background concentration making the cells hypersensitive, with the transmission of spurious signals which would cloud normal mental activity, trigger random thoughts and loss of concentration [37]. Hence, RF EMF-induced decrease in CaBPs expression as observed in the present study could decrease Ca^2+^ buffering capacity, leading to cell death.

RG has been used in oriental medicine for thousands of years with ginsenosides as its main bioactive component plays an important role in CNS [2, 3]. Ginsenosides can modulate the functions of many receptors and ion channels on neurons, including N-methyl-D-aspartate (NMDA) receptors [5, 6] and Ca^2+^ channels [38]. Inhibition of an increase in Ca^2+^ influx by RG has been noted in the hippocampal neurons subjected to oxygen/glucose deprivation [39]. RG has been suggested to act as a neuroprotectant by mediating the inhibition of Ca^2+^ influx through both NMDA receptor channels and L-type voltage-dependent Ca^2+^ channels, with the resultant reduction of intracellular free Ca^2+^ [39]. Inhibition of Ca^2+^ influx by RG has been reported in a glutamate (0.5 mM) toxicity model of cultured hippocampal neurons of rats [40]. Similar findings in cultured hippocampal neurons have also been noted by various studies [41–43]. Similarly, in the present study, the ginseng-treated group did not show harmful effect from EMF exposure, which also supports the previously mentioned findings further consolidating its radioprotective capabilities.

With the ever-increasing growth of mobile communication, an increase in EMF density has occurred which can influence neuronal function, neurotransmitter release, neuronal survival, and learning and memory [15]. The present study noted loss of neurons in the CA as well as in the granular layer after EMF exposure. Severe loss of dendritic arborization was noted. Similar to our study, neuronal damage and cell loss in the CA area of the female rat hippocampus was noted after exposure at 900 MHz [16, 17], while the prenatal exposure revealed decrement in the number of granule cells in the dentate gyrus [44] by cell death and the inhibition of the differentiation of neural stem cells into neurons [45]. Different hippocampal regions showed different susceptibility to injury. Decrement of CaBPs IR in the hippocampal subfields (CA1, CA3, and dentate gyrus) is to be strongly noted as this part of the brain is an important link in the hippocampal trisynaptic circuit related to memory and learning [46]. Loss of interneurons in the CA areas is also particularly important as it is associated with complex cognitive, mnemonic, and emotional processes through their connection to the prefrontal region, for example, to the medial and orbital areas [47]. 

The hippocampus is an important area of interest as it is the primary target for research in the molecular and cellular mechanism of memory and information storage through its synaptic connections [48]. The present study elucidated the preventive effect of RG in regard to CaBP IR in the hippocampal subfields after RF exposure. A decrease in the CaBP IR in the hippocampal subfields in the present study denotes a decrement in the Ca^2+^ buffering capability which might ultimately lead to cell death, while loss of neurons in the CA regions and dentate gyrus further strengthens the reasoning behind the increased Ca^2+^ influx. In the same model the ginseng-administered group did not show any loss of CaBP IR or neuronal loss due to RF exposure. Various studies have also noted the beneficial effects of ginseng in the hippocampus [49–52], which supports the findings of the present study. Oral administration of RG prolonged the survival of newly generated cells, and long-term survival was noted to be present in the hippocampal subfields [53], which rightly could be the contributing factor behind the negation of the effect of EMF in the ginseng-treated group. Ginseng has been reported to inhibit NMDA-induced increase in intracellular free Ca^2+^, and the inhibition of homocysteine-induced elevation of intracellular free Ca^2+^ has been noted as well. Memory test could be helpful in further consolidating the present findings. Ginseng is also known to have anti-inflammatory and antiapoptosis activities which might be the reason it did not affect CaBP IR. A TUNEL experiment might be of help to prove this point. Another possible explanation could be the promotion of cell genesis by RG, which is also strengthened by our findings (data not shown) that are similar to the findings that noted increased brain-derived neurotrophic factor (BDNF) expression in the hippocampus [54]. BDNF has been well documented to participate in the survival, differentiation, and maintenance of the functions of specific types of neurons in the central nervous system [55]. It also influences synaptic strength and neuronal plasticity [55] and both neurotransmitter and neurotrophic factor synthesis [55]. Increase in potential health risks due to excessive use of mobile communications has generated interest in determining an effective radioprotector that could negate the negative effects of RF exposure. Although a perfect radioprotector has not yet been discovered, RG extract might emerge as a nontoxic radioprotective agent that can maintain Ca^2+^ homeostasis in the hippocampus.

In summary, the present study showed the protective effect of RG against RF exposure by maintaining the CaBP IR involved in the buffering of Ca^2+^, the lack of which might cause severe damage leading to tissue degeneration. However, the proper mechanism is yet to be unveiled. Further studies with protein assay test, western blots, and electrophysiological recording might help to shed more light on it. In order to thoroughly understand the beneficial effect of RG against RF exposure, much research is still needed to further consolidate RG as a universally accepted radioprotective agent.
